# Supplementary material for: Are carbon emissions trading and green financial instruments synergistic? -Comprehensive quantitative research based on content analysis
Source: PLoS One. 2024 Mar 7;19(3):e0298601. doi: 10.1371/journal.pone.0298601 (PMC10919601; doi:10.1371/journal.pone.0298601)
Supplement: S1 Data — (ZIP) [file pone.0298601.s001.zip › Data/地级市Z_F工作报告.zip!/░▓╗╒/┘±╓▌╩╨/└┤╫╘╣1⁄2╓┌║┼í╛┬φ┐╦╩2╛▌═°í┐_2017.docx]

各位代表：
　　现在，我代表市人民政府，向大会报告政府工作，请予审议，并请市政协委员和其他列席人员提出意见。

****一、2016年和本届政府工作回顾****

　　2016年，在省委、省政府和市委的坚强领导下，市政府紧紧依靠全市人民，全面贯彻党的十八大和十八届三中、四中、五中、六中全会精神，深入学习贯彻习近平总书记系列重要讲话特别是视察安徽重要讲话精神，坚持稳中求进、进中求优、优中求快的工作总基调，紧盯人民群众关心关注的热点问题、社会各界反映强烈的焦点问题、当前工作中亟待解决的重点问题、制约亳州长远发展的难点问题，主动适应经济发展新常态，大力推进供给侧结构性改革，攻坚克难，奋力拼搏，圆满完成市三届人大七次、八次会议确定的各项目标任务，实现了“十三五”发展良好开局。
　　1-12月，在省统计局公布的24项主要经济指标中，我市有20项增速居全省前8位，其中12项居全省前5位、7项居全省前2位、2项居全省第1位，主要经济指标增速继续保持在全省前列。全年地区生产总值1046亿元，增长8.9%、超过全省平均水平；财政收入147亿元、增长13.2%，增速居全省第1位；固定资产投资874.9亿元、增长14%；社会消费品零售总额492.1亿元、增长12.8%；城镇常住居民人均可支配收入25053元、增长8.4%，农村常住居民人均可支配收入10576元、增长8.6%。
　　（一）“调转促”步伐进一步加快
　　战略性新兴产业发展迅速。制定出台药品技术转让研发和培育、市外知名企业并购本市药业企业、中药配方颗粒生产等系列政策，设立首批10亿元药品技术转让和研发专项投资基金，5家药业企业完成重组，中药配方颗粒生产基地获批建设并已组织开展生产和临床使用研究，300个药品技术转让申请已报省待批。新增规模以上中药工业企业14家、总数达178家，新通过GMP认证企业20家、总数达150家，国家中药材产品质量监督检验中心建成并试运行。安徽九方制药有限公司注射用牡荆素成功获得中药一类新药临床批件，填补了全省中药一类新药研发领域的空白。安徽济人药业有限公司疏风解毒胶囊单品种销售收入突破5亿元。战略性新兴产业实现产值297亿元、增长17.3%。现代中药产业集聚发展基地实现产值267亿元，新签约药业项目60个、总投资285亿元，新落户全国知名医药企业7家、总数达50家。联滔电子、创酷科技、国能蒙城生物质发电、海创新型节能建筑材料、信义光伏及玻璃制造等企业发展迅速，新一代信息技术、节能环保、新能源、新材料等产业取得长足发展。
　　传统产业加快升级。大力实施工业“四百计划”，新增规模以上工业企业134家，实现规模工业增加值272亿元、增长10%；实施重点工业项目200个，完成投资360亿元。规模以上食品工业实现总产值300亿元、增长10%。白酒产业实现产值100亿元、增长12%。古井集团生产销售逆势上扬，实现收入75亿元、增长10.7%，实现税收23亿元、增长14.7%。江淮安驰销售汽车4.5万台，实现产值23.4亿元、税收1.3亿元。新增中国驰名商标2件、省著名商标28件。
　　第三产业稳步发展。净增限额以上商贸流通企业124家、总数达727家，万达广场、蒙城红星美凯龙等一批商业综合体建成开业，中国物流亳州综合物流园、亳州皖新物流园等商贸流通项目开工建设，中药材流通追溯体系试点项目通过商务部初验。实现旅游总收入120亿元、增长19.4%，国家中医药健康旅游示范区已上报待批，新创建国家4A级旅游景区2个，培育乡村旅游精品线路7条，利辛县获评为全国十佳生态休闲旅游城市，谯城区获评为最美中国?生态（自然）旅游胜地和美丽乡村旅游胜地。加大住宅用地供应力度，持续稳定房地产市场，完成房地产开发投资231.4亿元，商品房销售550万平方米。客货运周转量、快递业务量分别增长3.8%和92%。
　　（二）稳增长根基进一步夯实
　　投资规模不断扩大。建立市本级工业项目规划建设审批五项促进机制，前期审批流程由282天压减至100天。全年实施省市重点项目397个，完成投资595亿元、增长17.4%；续建项目198个，完成投资420亿元、增长21.7%；新开工项目199个，完成投资175亿元；新谋划储备项目1340个，总投资3500亿元。投资80亿元的神农谷中药电商物流城、20亿元的九洲方圆制药、19亿元的外经钻石广场、19亿元的碧桂园平侯府、17.5亿元的碧桂园龙城等项目进展顺利，投资10.6亿元的司尔特肥业、10亿元的太安堂中药物流及人参加工等一批项目建成投产，引江济淮入亳、涡河高等级航道改造等重大项目前期工作扎实推进。落实中央专项建设基金项目21个、总额27亿元。推行政府和社会资本合作PPP模式，投资17.6亿元的谯城区“产城一体化”项目被评为国家第三批PPP示范项目。
　　精准招商成效显著。全年实际利用市外内资1400亿元、增长15%，新签约招商项目300个、总投资1165亿元，其中工业项目230个、总投资756.8亿元。投资58亿元的长白山制药产业园、40亿元的恩度食品药材深加工基地、35亿元的蒙城九九慢城杜仲良种产业化等32个10亿元以上项目签约落户。成功举办2016年国际（亳州）中医药博览会暨第32届全国（亳州）中药材交易会，签约招商项目48个，总投资345.2亿元。2016中国国际徽商大会期间，我市签约项目54个，总投资420.9亿元、占全省的16.9%。
　　园区建设深入推进。市经济开发区道西片区和道东1号地块征迁全部完成，交付净地3.1万亩；亳芜现代产业园区新建、改建道路14.6公里，水电管网等基础设施不断完善；市食品产业园签约项目18个、总投资20.7亿元。6个省级开发区实现产值235亿元、增长15.2%，实现经营收入1080亿元、增长17.6%，完成财政收入35亿元、增长40%。
　　要素保障持续加强。扎实推进农村土地综合整治，争取土地增减挂、工矿废弃地复垦、新增建设用地指标共3.7万亩，计划指标总量居全省第1位，全省首家完成全域永久基本农田划定、土地利用总体规划调整完善。新增存款330.2亿元、贷款253.2亿元，分别高于上年130.9亿元、112.1亿元。保险业保费总收入56亿元、赔（给）付金额22亿元。“532”小额贷款保证保险累计发放1.5亿元，“4321”新型政银担业务在保余额29.3亿元，小微企业续贷过桥资金周转贷款48.2亿元、周转率20.5。新引进各类高层次人才30人，培育高技能人才2156人。
　　（三）农业现代化水平进一步提升
　　加快建设现代农业产业体系。全年粮食总产94.8亿斤，建设粮食绿色增产示范片45个、优质专用品牌粮食生产基地155万亩、中药材标准化种植基地22.5万亩，新增设施蔬菜种植面积8万亩。新创建畜牧绿色低碳健康养殖示范场56个，规模化养殖比重达82%，比上年提高2个百分点。大力发展农产品加工业，产值超亿元农产品加工企业115家，规模以上农产品加工业实现产值660亿元。新增“三品一标”认证产品62个，“涡阳大豆”获批国家地理标志产品。利辛县、谯城区被列为省级现代生态农业产业化示范县区。
　　加快建设现代农业生产体系。新增各类农机2.7万台套，农机销售额24.4亿元、是上年的8倍，市县共补贴农机购置资金13.2亿元、是上年的22.2倍。新增农机总动力118.5万千瓦，总量985.4万千瓦、居全省第1位。西淝河等沿淮洼地应急工程、6条中小河流治理工程全面建成，淮干一般堤防加固、港河泵站、病险涵闸除险加固等工程加快建设，完成农田水利基本建设投资13.2亿元，利辛县荣获省“江淮杯”一等奖。争取“十三五”期间高标准基本农田建设指标391万亩、资金39.1亿元，建设高标准基本农田113.4万亩，新增耕地3.2万亩。谯城区获批为全国首批农村产业融合发展试点示范区。新建高大平房仓35栋、仓容22万吨，维修加固老旧粮库315栋、仓容67万吨。
　　加快建设现代农业经营体系。新增市级以上示范合作社92家、家庭农场95家，全市适度规模经营面积394万亩。培育现代生态农业产业化联合体52个，农机社会化服务组织发展到1280家。全市农产品电子商务交易额突破10亿元。农村土地承包经营权确权登记颁证工作全面完成，三县一区农村产权交易中心初步建成，涡阳县农村承包土地经营权抵押贷款发放1.4亿元、居全省试点县第1位。
　　（四）改革创新活力进一步释放
　　供给侧结构性改革扎实推进。出台落实供给侧结构性改革“1+4”系列政策文件，清理“僵尸企业”8家，关闭刘店煤矿、退出产能150万吨，安置职工2002人。鼓励农民退宅进城购房，全面停止新建还原安置房，中心城区货币化安置比例达60%以上，商品房去化周期控制在合理区间。争取省代发置换债券69.4亿元并全部置换完成。完成省阶段性降低社会保险费率任务，减轻企业用工、用能、物流等成本3亿多元。
　　“放管服”改革实现突破。在全省率先编制完成公共服务清单和中介服务清单，梳理市级公共服务事项1321项，保留中介服务事项204项、取消26项、规范37项。优化“互联网+政务服务”，网上办事大厅累计办结申办件286.7万件，满意率99.9%；“政企直通车”微信平台办理企业反映问题1602个，满意率95%；“我要贷款”平台发放贷款37亿元，“税融通”发放贷款15亿元；“我家亳州”APP累计下载158万人次。设立市县不动产登记中心。建成市县一体化公共资源电子交易平台。商事登记实现“五证合一、一照一码”。
　　重点领域改革持续深化。规范预算管理，盘活财政存量资金14.9亿元。全面推开营改增试点，试点企业1.6万户，国税、地税业务办理互联互通，在全省率先实现“一窗办两税”，减免地方各项税收21亿元，办理出口退税2.8亿元。新增IPO报备企业1家、“新三板”挂牌企业8家、区域股权交易中心挂牌企业86家，实现直接融资184.7亿元、是上年的3.4倍。建安集团资产规模增至1123亿元、居全省第2位，新增融资397亿元、到位287.6亿元。药都银行资产规模达404亿元，营业收入和利润居全省农商行系统第1位。中小学、幼儿园教师无校籍管理改革被列为全国乡村教师队伍建设优秀案例，医药卫生体制综合改革进展顺利，市县两级妇幼保健和计生服务机构整合工作全面完成，机关事业单位养老保险制度改革有序展开，3个国有林场改革工作全面启动。
　　大众创业万众创新蓬勃发展。深入开展“亳州老乡，请您回家”和助力青年学子创业活动，成立市“请您回家助您创业”办公室，设立1500万元创业扶持专项资金，4375个创业线索已成功转化3217个。出台“互联网+创业示范园”鼓励政策和促进农民工进城就业的若干意见，筑梦社区、青年创客空间分别入驻企业209家、70家，发放创业担保贴息贷款7.9亿元，新增市场主体4.7万户、增长23%。高新技术产业实现产值350亿元、增长22%；新认定高新技术企业24家、授权发明专利155件，新增省级企业技术中心10家、省级新产品8件。
　　开放发展空间不断拓展。全年实际利用外商直接投资7.2亿美元、增长10%，实现进出口总额5.3亿美元、增长5%，新增进出口实绩企业23家、总数达128家。推动企业“走出去”，新培育外经主体5家，组织开展境外经贸活动4次。
　　（五）新型城镇化建设进一步提速
　　突出规划引领。扎实推进全域空间统筹规划和“城乡建设一张图”编制工作。编制完成新型城镇化发展规划、综合交通规划、高铁站片区规划和中心城区近期建设规划、空间特色规划，以及公厕、充电桩、菜市场等32个专项规划，整合完成43个单元规划。编制完成所有行政村村庄布点规划和涡阳县“多规合一”试点总体规划。
　　推进城乡建设。在市中心城区实施城建项目274个、完成投资128.1亿元。市垃圾焚烧发电厂、安置还原小区D区、药都大道棚户区改造、华佗广场一期改造等项目投入使用，50条道路、文化公园、养老服务中心等开工建设。中心城区日供水能力提高到12万吨，16条主次干道部分路段弱电下地工程顺利实施，新投入纯电动公交车150辆，建设公交首末站3个。新改建国省干线公路194.5公里，建成农村道路畅通工程3655公里，绕城快速通道西线、南线、北线基本贯通。三县县城建设步伐加快，累计实施项目211个、完成投资63.6亿元，涡阳县南部新区发展框架全面拉开，蒙城县城市森林公园西片区项目加快建设，利辛县新建、续建城市道路27条。蒙城县、利辛县获评为国家园林县城。2015年度31个省级中心村建设、2016年度52个省级中心村和37个乡镇政府驻地建成区整治建设全部完成，利辛县美丽乡村整县推进工作有序展开。
　　强化科学管控。理顺城市管理体制，成立市城市管理行政执法局。数字城管系统受理事项24.8万件，办结率96.3%。拆除中心城区违法违规户外广告20048块（处、个）、电话报刊亭240座，完成魏武大道、希夷大道楼体立面整治工作，利用市政基础设施小修机制修复破损设施1450处，理顺中心城区路灯管理体制，建成区路灯亮灯率98%，整治背街小巷35条，改造老旧小区15.8万平方米，新建改建公厕50个，住宅小区物业服务覆盖率85%。集镇交通、卫生、建设、经营“四大秩序”整治和农村清洁工程、“三线三边”环境治理深入推进，清理垃圾22万吨、河道4800公里。
　　（六）生态环境进一步优化
　　“地净、路畅、水活、天蓝、林拥城”工程持续推进。中心城区“五车”实现信息化监管，城市干道机械化清扫率90%以上。魏武大道南段等6条断头路顺利打通，建安路涡河隧道、汤王大道涡河隧道开工建设。宋汤河四期、凤尾沟二期工程全部完工，“一涵四闸”工程投入使用，中心城区涡河以南、铁路以西水系全面贯通，水质明显改善。秸秆禁烧成效显著，全年卫星监测火点数由上年的53个下降到2个，秸秆综合利用率85%。关闭无证自办商品混凝土搅拌站88家、其中已拆除31家，列入市政府目标考核的建筑工地扬尘治理达标率100%，淘汰黄标车和老旧车8360辆，PM10和PM2.5年均浓度较上年分别下降4.9%、4.3%，主要污染物减排超额完成省下达任务。“林拥城”项目完成投资2.3亿元、栽植苗木6000多亩、建成环城林带13公里，建成城市绿道52公里，国家园林城市创建工作稳步推进。
　　生态建设成效显著。千万亩森林增长工程顺利收官，人工造林9.8万亩，建设绿色长廊764公里，全市森林覆盖率18.4%。利辛县获评为全国绿化模范县。市污水处理厂、南部新区污水处理厂扩建工程开工建设，中心城区污水集中处理率95.7%，全市日污水处理能力30万吨。83家规模化养殖场完成污染综合治理任务，关闭或搬迁禁养区内规模养殖场60家，市县11条（处）黑臭水体整治工程全面启动，宋汤河沿岸39处排污口实现截污，涡河出境水质好于入境水质。
　　资源利用效率持续提升。全面落实最严格水资源管理制度，严守“水资源开发利用、用水效率、水功能区限制纳污”三条红线，万元GDP用水量下降6.2%。清理闲置低效用地2619亩，建成标准化厂房40万平方米。5个光伏电站并网发电、总装机规模200兆瓦，签约落地生物质发电项目9个。单位GDP能耗下降3%。
　　（七）和谐稳定局面进一步巩固
　　脱贫攻坚步伐加快。成立市扶贫局，编制贫困村贫困户帮扶措施大全，科学划分“四个清单”，配套出台光伏、教育、健康、特色种养、乡村旅游、社保兜底等系列政策。全年投入扶贫资金2.56亿元、是上年的8.2倍，其中市本级安排1.03亿元、是上年的20.6倍。在皖北地区率先实现农村五保分散供养标准、低保标准、扶贫标准“三线合一”，免除义务教育阶段贫困家庭子女作业本费和教辅资料费。建成户用光伏电站16080座、村级光伏电站187座。发放贫困户小额扶贫贷款1.4亿元，完成贫困户危房改造任务5110户、贫困村电网改造项目218个，开工建设贫困村道路1011.3公里，饮水安全工程、高标准基本农田、宽带网络和4G网络实现贫困村全覆盖。市脱贫攻坚信息服务平台建成使用，日均回访贫困户150户。19个易地扶贫搬迁安置点全部开工建设。为35个贫困村各招聘了1名医学专业本科毕业生。预计全年出列161个村、脱贫84150人。
　　民生工程顺利实施。32项民生工程投入资金125.8亿元，全面完成年度任务。基本建成各类保障性安居工程27006套。新增城镇就业3.8万人，转移农村富余劳动力10.7万人。新增养老服务机构床位1000张，五保集中供养能力75%。为9.8万名80岁以上老人发放津贴1562.4万元。机构集中养育孤儿、散居孤儿基本生活费标准每人每月提高300元，分别达1300元、900元，累计发放2819.5万元。救助流浪乞讨人员3700人次。解决13.9万农村居民饮水安全问题。17个乡镇食品检验室、33个社区和农产品批发市场食品快速检测室全部建成。
　　社会事业全面发展。新建、改扩建中小学42所，建成公办幼儿园40所，完成“村小提升工程”项目200个，改造薄弱学校431所。市精神病医院投入使用，市人民医院二期工程建成，华佗中医院新院主体工程完工，新建二级以上民营医院10家，所有乡镇卫生院和社区卫生服务中心均设置中医科。人口自然增长率8.6‰。市体育公园开工建设，新建市中心城区室外游泳池10个、社区公共体育场地170片、室外健身器材326套，成功举办市第二届运动会，我市被授予“国家健身气功五禽戏传承地”称号，校园五禽戏创编结题报告通过国家体育总局评审。公共文化场馆全部免费开放，广播村村通实现自然村全覆盖。尉迟寺遗址保护规划获国家文物局批准实施。
　　社会治理不断加强。扎实推进“平安亳州”建设，建成市级视频数据平台，刑事案件发案下降18.2%、治安案件下降24.6%。网上信访投诉平台正式开通，517件重点信访事项全部化解。137个省市挂牌安全隐患已整改136个，排查一般安全隐患20519个、整改率98.6%。消防监督服务和灭火救援水平有效提升。全面启动“七五”普法规划，覆盖城乡的公共法律服务体系实体平台基本建成。精神文明建设扎实推进，13人入选“中国好人榜”，24人当选“安徽好人”及获提名奖。国防教育、国防动员、人民防空工作进一步加强，军民融合深度发展。民族宗教、妇女儿童、档案、地方志、红十字、残疾人、慈善公益事业健康发展。统计、外事、侨务、对台、供销、气象、地震、防灾减灾、应急管理、公积金管理、新闻出版、无线电管理、食品药品监管和关心下一代工作取得新进展。
　　（八）政府自身建设进一步加强
　　扎实开展“两学一做”学习教育，健全市政府党组工作规则、重大事项向市委请示报告等制度，建立经济工作、项目建设、环境保护和安全生产四个“双调度”制度，完成年度岗位责任目标任务676项，整改“四个点问题”中涉及政府工作问题清单事项690项，有效保障了省委、省政府和市委各项决策部署的快速落实。坚持依法行政，健全重大事项决策程序和合法性审查程序，提请审议地方法规草案2件，重大事项合法性审查率100%。办理人大议案建议200件、政协建议案和提案358件，满意率均在98%以上。畅通群众沟通渠道，依法公开政务信息10.8万条，通过人民网网友留言、市长热线、政风行风热线、沟通—政务零距离等渠道受理群众诉求16万多件，办结率96%以上。贯彻落实中央八项规定精神和省市“三十条”规定，全面整改政府系统承担的中央巡视“回头看”、省委巡视组巡视和国务院第三次大督查反馈问题，着力强化行政监察，全力推进审计监督、制度规范全覆盖，查处政府系统违法违纪案件174件，政府系统党风廉政建设取得新成效。
　　各位代表，2016年各项任务的完成，标志着本届政府工作的圆满收官。回首过去六年，面对各种复杂严峻的考验，在省委、省政府和市委的坚强领导下，我们紧紧依靠全市人民，保持战略定力，坚定发展信心，埋头苦干实干，全市经济社会进入了加速崛起、跨越赶超的崭新阶段。
　　六年来，我们聚焦扩量提质，致力转型升级，综合实力快速增强。地区生产总值连续迈上5个百亿元台阶，突破1000亿元，是2010年的2倍。全部财政收入是2010年的3.5倍，在全省排名提升4个位次。固定资产投资是2010年的3.2倍。社会消费品零售总额是2010年的2.2倍。银行存贷比由44.9%提高到65.1%。三次产业结构由26.7：37.4：35.9优化为19.5：38.9：41.6，规模工业企业突破1000家，总产值突破1000亿元，工业化率提高2个百分点，战略性新兴产业增加值占比由15.4%提高到27.5%。6个省级开发区入园企业由360家增加到1350家，产值由25.2亿元提高到235亿元。
　　六年来，我们聚焦深化改革，致力扩大开放，创新活力充分激发。“互联网+政务服务”、商事制度改革、农村综合改革走在全省全国前列，“放管服”“调转促”积极推进，各类市场主体由11万户增加到25万户，民营工业增加值占规模以上工业比重由47.9%提高到73%，民间投资占固定资产投资比重由57.2%提高到64.5%。累计利用市外内资5486亿元、年均增长29.5%，进出口总额26.5亿美元、年均增长14.2%，实际利用外商直接投资30.6亿美元、年均增长28.7%。累计实现高新技术产业增加值75亿元、年均增长18.9%。
　　六年来，我们聚焦协调推进，致力统筹发展，城乡面貌焕然一新。城镇化率达38%，年均提高1.5个百分点，增速居全省第1位。中心城区建成区面积扩大到65平方公里，集聚人口60万人。三县县城建成区总面积扩大到94平方公里，集聚人口79.5万人。市县乡村交通大幅改善，泗许高速、济祁高速亳州段建成通车，S307亳州段全线升级为一级公路，新改建农村公路5784.5公里，改造公路危桥636座。美丽乡村建设在全省考核中连续两个年度位居先进行列。
　　六年来，我们聚焦民生保障，致力共建共享，人民生活持续改善。民生支出累计1107亿元、占财政总支出的84.5%，新建保障性住房16.2万套，改造农村危房3.7万户，城乡基本养老、基本医疗保险和居民大病保险实现全覆盖，54.7万人稳定脱贫。学前教育毛入学率、初中毕业升学率、普通高考本科达线率分别为90.3%、97.2%和51.3%，较2010年分别提高32.4、25.5和14.1个百分点；职业教育进一步发展，在校学生达7.3万人，是2010年的1.4倍。累计新增城镇就业33.2万人，转移农村富余劳动力88.9万人，解决435.5万农村居民和19.9万名师生饮水安全问题。城镇和农村常住居民人均可支配收入分别是2010年的1.6倍、2.3倍。
　　六年来，我们聚焦工作重点，致力长远发展，干成一批大事要事。首次荣膺全国双拥模范城、安徽省文明城市，三次荣获“全国粮食生产先进单位”称号，连续五年荣获“全省人口与计划生育工作先进市”称号，成功创建全省首个“小麦亩产千斤市”和“吨粮市”，顺利跻身国家智慧城市试点市、国家创建新能源示范城市试点、全省首批战略性新兴产业集聚发展基地。尤其是，商合杭高铁开工建设，亳州机场选址获批，板集电厂并网发电，亳州学院挂牌招生、结束了我市没有本科院校的历史，“现代中医药及健康产业亳州专板”正式开板，极大地提振了全市人民干事创业的精气神。
　　各位代表，六年的成绩来之不易，六年的成就令人振奋！六年来，我们砥砺奋进，铿锵前行，一步一个脚印，一年一大台阶，各项事业都取得了显著成绩。这些成绩的取得，是省委、省政府和市委正确领导的结果，是历届政府、历任老领导老同志接力奋斗的结果，是市人大和市政协监督支持的结果，是全市635万人民团结拼搏的结果。在此，我代表市人民政府，向全体人大代表、政协委员，向全市各条战线上的工人、农民、知识分子、干部、人民解放军指战员、武警官兵、政法干警以及社会各界人士，致以崇高的敬意！向所有参与亳州建设的各位投资者和关心支持亳州发展的各界朋友，表示衷心的感谢！
　　六年的奋斗历程和全市人民的伟大实践，给予我们宝贵启示：办好亳州的事情，必须坚持解放思想不动摇，以新理念引领新发展，不断向改革开放要动力，向创新创业要活力，向特色优势要竞争力；必须咬住发展不动摇，以“见第一就争、见红旗就扛”的勇气担当，快马加鞭、苦干实干，比别人干得更多、比别人干得更实、比别人干得更拼；必须坚定绿色发展不动摇，像爱护眼睛一样保护生态，像对待生命一样对待环境，坚持走可持续发展之路；必须致力改善民生不动摇，坚持以人民为中心的发展思想，时刻把群众的安危冷暖放在心上、扛在肩上，让发展更有温度、让幸福更有质感！
　　与此同时，我们也清醒地看到，我市经济社会发展仍存在一些突出矛盾和问题：一是发展不足，结构不优，经济总量较小、工业基础薄弱、农业产业化水平偏低、现代服务业发展不快的状况仍未根本改变，大项目特别是拉动作用大的工业项目不多，项目建设对经济的支撑作用仍待提高。二是创新能力不足，对外开放程度不够，科技研发投入较少，高层次人才紧缺。三是城镇化水平不高，社会事业欠账较多，城乡道路、环境卫生、污水处理等基础设施存在不少短板，安全生产、污染减排等领域还存在一些亟待整改的问题。四是少数干部担当精神不足、争先意识不强、解决问题的办法不多，不愿为、不会为、不敢为、作风不实等问题还不同程度存在，政府系统机关效能仍需进一步提高。所有这些问题，必须高度重视，切实增强忧患意识和危机意识，采取有力措施加以解决。

****二、今后六年的奋斗目标和主要任务****

　　今后六年，是“深入践行五大发展理念，奋力走在皖北振兴前列”的关键时期，是我市决胜全面小康的攻坚阶段。国务院批准了促进中部地区崛起的“十三五”规划和中原城市群发展规划，省第十次党代会作出了加快皖北地区“四化”协调发展、支持亳州建设省际交汇区域中心城市的决定，市第四次党代会明确了建设“新兴工业强市、现代农业强市、文化旅游强市、新型智慧城市、健康养生城市”的奋斗目标，描绘了亳州发展的美好蓝图。我们要抢抓战略机遇，牢牢扭住发展第一要务不动摇，矢志不渝、锐意进取，奋力谱写亳州改革开放和现代化建设的新篇章。
　　今后六年经济社会发展的指导思想是：全面贯彻党的十八大和十八届三中、四中、五中、六中全会以及即将召开的党的十九大精神，深入学习贯彻习近平总书记系列重要讲话特别是视察安徽重要讲话精神，统筹推进“五位一体”总体布局和协调推进“四个全面”战略布局，坚持稳中求进、优中求快、奋发有为的工作总基调，坚定地用新发展理念统领发展全局，适应把握引领经济发展新常态，贯彻落实“五大政策”，以“深入践行五大发展理念，奋力走在皖北振兴前列”为总要求，以提高发展质量和效益为中心，以推进供给侧结构性改革为主线，以实施五大发展行动计划为总抓手，积极扩大有效需求，着力实施创新驱动发展战略，紧盯“四个点问题”，坚持问题导向、目标导向、服务导向、务实导向，全面做好稳增长、促改革、调结构、惠民生、防风险各项工作，坚定地在践行五大发展理念中闯出新路，不断开创奋力走在皖北振兴前列新局面。
　　今后六年经济社会发展的奋斗目标是：
　　——综合实力显著增强。主要经济社会发展指标领先皖北、在全省位次前移，地区生产总值达1850亿元；固定资产投资、财政收入、规模工业增加值在2016年的基础上翻一番，分别达到1700亿元、300亿元、500亿元；进出口总额突破8亿美元。
　　——质量效益显著提升。三次产业结构优化为12.5：41.5：46，工业化率达40%以上，战略性新兴产业和现代服务业比重明显提高，新兴工业强市、现代农业强市、文化旅游强市建设取得新成效。
　　——城乡建设显著加快。城镇化率提高到48%，中心城区辐射带动作用和县域经济实力持续增强，新型智慧城市、健康养生城市建设加快推进，美丽乡村、美丽集镇建设扎实推进，城乡统筹、一体化发展水平显著提升，百万人口大城市基本建成。
　　——生态环境显著改善。“地净、路畅、水活、天蓝、林拥城”工程取得显著成效，空气质量优良率达80%，污染治理、节能减排和生态保护修复取得明显成效，实现地更净、路更畅、水更清、天更蓝、城更美。
　　——生活水平显著提高。居民收入达到全省平均水平，城乡居民收入差距逐步缩小。实现贫困人口全部脱贫。就业、社保、医疗、住房、食品药品安全等公共服务体系更加健全，基本公共服务更加均等。
　　今后六年经济社会发展的主要任务是：
　　（一）实施创新发展行动，全力培育经济增长新动能。深入实施创新驱动发展战略，积极对接“三重一创”建设工程，扎实推进产业创新升级，大力支持实体经济发展，全力推动大众创业万众创新，集聚创新发展新动能。大力实施创新平台建设工程，培养招引各类“高精尖缺”人才，打造一批创业园区、技术转移中心、创客载体，力争规模以上工业企业研发机构覆盖率达30%。大力实施创新创业主体培育工程，力争全市高新技术企业达100家以上。现代中药产业实现“双千亿”目标，农产品加工业产值突破1200亿元，服务外包产业加快发展，文化旅游和健康养老产业成为新的经济增长极。深入推进行政体制、农业农村、财税金融、国资国企、住房制度等重点领域改革。
　　（二）实施协调发展行动，全力构建统筹联动新格局。围绕“省际交汇区域中心城市”定位，大力实施城乡规划、建设、管理提升工程，加快铁路、公路、水利、航道、航空、电网、气（油）管线、信息网络、物流通道设施建设，打造百万人口大城市。加快发展县域经济，支持三县一区城市提质扩容、产业错位发展，确保县域经济总量突破1530亿元，力争有1个县区进入全省十强县。大力实施美丽乡村建设工程，扎实推进美丽集镇和中心村建设，加快农村基础设施改造提升。深入开展精神文明建设，创建全国文明城市、国家卫生城市、全国未成年人思想道德建设工作先进城市。
　　（三）实施绿色发展行动，全力建设生态宜居新家园。打造绿色环境，发展绿色产业，倡导绿色生活，建设人水和谐、绿色共享的美丽家园。加强生态环境保护，创建国家园林城市、国家环保模范城市，全市绿化覆盖率达38%，城市建成区绿地率达32%。加强资源高效利用，发展可再生能源和清洁能源，建立循环产业体系，加快绿色低碳发展。建立健全资源有偿使用和生态补偿机制，严格落实领导干部生态环境损害责任追究制度，强化大气、水、土壤污染治理，推进“五水共治”和“减排、压煤、降尘、治车、禁烧”工程，建成区污水收集率和处理率均达99%以上。
　　（四）实施开放发展行动，全力拓展经济发展新空间。深入实施招商引资提质增效工程，确保利用市外内资年均增长15%左右、外商直接投资年均增长10%左右。深入实施综合交通体系建设工程，全面完成商合杭高铁建设，助推亳蚌、淮阜城际铁路开工建设，加快推进亳州机场、亳蒙高速、淮阜高速、涡河高等级航道建设，力争高速公路里程达356公里、国省干线里程达1340公里、农村道路畅通工程达12800公里。深入实施外贸主体培育壮大工程，依托“一带一路”，加快建设对外开放平台，推动设立海关正式机构。深入实施园区转型升级工程，创建1个以上国家级开发区。
　　（五）实施共享发展行动，全力增进人民生活新福祉。坚持“六个精准”，紧盯“两不愁三保障”目标，深入推进脱贫攻坚工程，确保全面小康路上不落一人。深入推进就业创业促进工程，新增城镇就业15万人以上，转移农村劳动力35万人以上。深入推进社会保障全覆盖工程，认真办好人民满意的教育，着力打造城市社区15分钟健身圈、城市15分钟文化圈、农村5公里文化圈。加强法治亳州、平安亳州、信用亳州建设，大力推进社会管理信息化建设、食品药品“智慧监管”、安全生产“铸安”行动，着力促进军民融合深度发展。

****三、2017年重点工作****

　　今年是实施“十三五”规划的重要一年，是供给侧结构性改革的深化之年，是新一届政府的开局之年，做好各项工作意义重大。全市经济社会发展的主要预期目标是：地区生产总值增长9%左右，财政收入增长10%左右，固定资产投资增长14%左右，规模工业增加值增长12%左右，社会消费品零售总额增长12%左右，进出口总额增长5%左右，城镇常住居民人均可支配收入增长9%左右，农村常住居民人均可支配收入增长10%左右，节能减排、大气和水污染防治完成省政府下达的年度目标任务。
　　为实现上述目标，重点要抓好10个方面的工作：
　　（一）突出工业主导地位，加快建设新兴工业强市
　　发展优势产业。围绕现代中药产业“双千亿”目标，大力实施“369”行动计划，实现医药制造业总产值320亿元、增长18%，新增规模以上药业企业15家。加快推进现代中药产业集聚发展基地建设，完成固定资产投资95亿元、增长15%，实现产值310亿元、增长16%，引进中成药大品种3—5个，完成300个以上药品的技术转让受理工作，新通过GMP认证企业15家以上。深入推进中药配方颗粒生产基地建设，争取3—5家企业开展省中药配方颗粒生产和临床使用研究。大力发展白酒产业，实现产值120亿元、增长20%。大力发展电子信息、汽车制造、农机装备、鞋服箱包等劳动密集型产业，实现产值50亿元、增长8%。大力发展食品制造产业，继续实施“百家龙头企业提升工程”，规模以上农产品加工企业突破500家、实现产值700亿元，市食品产业园入驻企业20家以上，县区每个食品产业园新入驻企业5家。
　　壮大产业规模。扎实推进工业“三百工程”，加快实施100个在建项目、100个亿元项目、100个技改项目，完成工业投资400亿元、增长15%，完成技改投资140亿元、增长15%。扎实推进工业“123工程”，加快培育100家小巨人企业、200家成长性小企业、300家基础较好的小微企业，鼓励企业做大做强、做专做精，新增规模以上工业企业100家、销售收入亿元以上企业200家、5亿元以上企业25家，形成工业企业梯次成长格局。
　　提高创新能力。积极引导企业加大研发投入，支持企业引进转化新技术、新产品、新工艺，鼓励企业与高校院所合作组建技术转移中心，新增省级企业技术中心5家以上、专精特新中小企业10家、新产品6个。实施质量兴市和品牌强市战略，培育申报中国驰名商标1件、安徽名牌产品4个。大力培育引进创新主体，认真做好招才引智工作，认定高新技术企业15家，引进各层次科技人才团队5个以上，新增授权发明专利160件。着力打造创新平台，推进国家农业科技园区建设，充分发挥国家中药材产品质量监督检验中心作用，打造全国一流、涵盖中药材全产业链的第三方检测公共服务平台。
　　（二）突出农业提质增效，加快建设现代农业强市
　　优化农业生产结构。大力实施“双千”增收工程，积极推进农业增产、农民增收，确保粮食总产稳定在96亿斤以上。优化农业种植结构，建设优质粮食生产基地150万亩以上，中药材种植面积发展到116万亩，蔬菜面积稳定在200万亩。发展生态循环牧业，新建绿色循环健康养殖示范场60个，创建省级以上规模畜禽养殖示范场3个，力争规模养殖比重达83%。提升休闲观光农业水平，新培育乡村旅游精品线路4条，新创建省级以上休闲农业与乡村旅游示范点1个。继续实施“绿色亳农”品牌培育计划，新增无公害农产品产地、绿色食品原料基地20个，新增“三品一标”认证产品60个。
　　激发农业发展活力。开展省级现代生态农业产业化示范县区创建，新创建示范主体30个、联合体20个。积极培育新型职业农民和农业经营主体，新增省级示范合作社6家、省级示范家庭农场12家。投资1.5亿元，新增粮食烘干设备72套、蔬菜水果冷藏库65座。健全土地流转信息服务平台功能，适度规模经营面积发展到400万亩。实施农村土地所有权、承包权、经营权分置改革，推广农村承包土地经营权抵押贷款试点经验，推行“532”小额贷款保证保险模式，缓解新型农业经营主体贷款难问题。
　　夯实农业发展基础。继续实施小型水利工程改造提升“5588”行动计划，推进芡河、阜蒙河、北淝河等中小河流治理和港河大型泵站改造工程，完成淮干一般堤防加固和陈桥闸、洺河闸、油河闸除险加固等工程。新开工建设高标准基本农田40万亩以上。支持农机合作社建设，新增省级以上示范社6家，建设标准化农机库棚300个。落实农机购置补贴政策，优化农机装备结构，力争主要农作物耕种收综合机械化水平达90%以上。推进农村电网升级改造，完成投资3.6亿元。创建全省人工影响天气标准化县区1个，建设气象防灾减灾标准化镇、村各8个。建设数字化示范粮库80个。
　　（三）突出三产转型升级，加快推进服务业跨越发展
　　加快发展全域旅游。坚持把旅游业作为我市经济发展的重要增长极，以创建国家中医药健康旅游示范区为契机，以开展“亳州文化旅游年”系列活动为抓手，围绕旅游精品线路、精品景区、优质业态，加快全域旅游目的地建设，把我市打造成为皖北旅游中心城市，实现旅游总收入160亿元、增长35%。编制完成市全域旅游规划，加快重点文化旅游项目建设，新建、续建文化旅游项目23个、总投资222亿元、年内完成投资59亿元。举办亳州国际雕塑大赛，建成市文化馆、郑店子温泉度假区及游船码头等项目，开工建设北关历史街区保护与利用工程、市游客集散中心，实施花戏楼、中华酒谷创建5A景区和新四军四师红色旅游景区等提升工程。加快涡河文化旅游产业带、谯城区现代农业示范区“亳药花海休闲观光大世界”、老子生态园、庄子文化旅游区、西淝河生态休闲度假区等景区建设，实现三县一区串点成面、联动发展。
　　持续发展金融业。稳步推进金融机构网点建设，增设网点5个。不断创新金融产品和服务，扎实推进“4321”政银担合作、“税融通”和“我要贷款”平台等工作，新增信贷投放150亿元以上。完善多层次资本市场体系，支持企业加快上市步伐，新增“新三板”挂牌企业5家、区域股权交易中心挂牌企业10家，继续办好“现代中医药及健康产业亳州专板”，实现直接融资80亿元。切实发挥保险保障功能。
　　繁荣发展商贸流通业。优化大型商业网点总体布局，加大商贸流通企业培育力度，新增限上企业80家以上。积极推动大型商贸流通企业营销网络向农村延伸，完善“新网工程”网络，新建和改造农家店120家。加快推进新发地农副产品物流批发市场及冷链物流园、圆通速递皖北区域中心、涡阳中农批皖北农贸电商国际物流园、蒙城天河广场等重点商贸流通项目建设。加快推进神农谷中药电商物流城、康美（亳州）中药物流园等项目建设，着力打造全球最大的中药材集散地和中药生产销售集聚地。
　　大力发展新兴服务业。加快建设新型智慧城市，优化升级网上办事大厅，推广“我家亳州”APP平台运用，重点建设市政务云平台、大数据双创中心，发展云计算和大数据产业，开发利用视频数据资源，推动信息技术更好服务经济升级和民生改善。落实服务外包产业发展扶持政策，启动“云上神州”大数据产业园建设，完善硬件配套设施，引进数据处理中心、呼叫中心等服务外包企业5家以上，创建省级现代服务业集聚区。鼓励社会资本投资发展养老服务业，促进农村敬老院转型升级，力争15%的敬老院转型为区域性养老服务中心。加快推进医疗卫生与养老服务相结合，市级和每个县区分别建成2家以上医养结合机构，新增各类医疗护理型床位1000张以上。
　　（四）突出项目引领带动，加快蓄积经济发展后劲
　　强力推进项目建设。坚持把项目建设作为第一抓手，认真落实“四督四保”和“双调度”制度，严格兑现项目推进奖补政策，新建、续建重点项目500个以上、总投资2000亿元以上、年度投资600亿元以上。充分发挥重大项目的牵动作用，规范执行工业项目前期工作流程，严格约定开竣工时间，做好跟踪保障服务，确保建成九洲方圆制药、修正中药生产基地、信利新能源100兆瓦光伏电站、中国亳州现代服装产业基地等一批亿元以上重点产业项目，开工建设长白山制药产业园区、民生药业产业园、鸿路装配式建筑生产基地等项目。积极跟踪国家、省重点投资领域和方向，重点谋划一批产业项目和基础设施项目，新谋划项目1000个以上、总投资3000亿元以上。加强中央一般转移支付和专项转移支付的申报争取工作。
　　精准推进招商引资。坚持把招商引资作为第一要事，继续实行领导带头招商制度，积极开展节会招商、驻点招商、专业招商、以商招商，探索开展委托招商、网上招商，加快推动异地商会建设，全年利用市外内资增长15%以上。重点抓好现代中药、食品制造、服务外包、文化旅游等产业招商，引进亿元以上工业项目120个，其中亿元以上现代中药项目15个、食品加工项目8个。充分发挥园区招商引资的引擎作用，每个省级开发区至少引进10亿元以上项目1个、5亿元以上项目5个。建立招商线索推进落地联席会议制度，完善招商引资考核办法，全力营造大招商、招大商的浓厚氛围。
　　加快推进园区转型。坚持把园区作为经济发展的主阵地，加速提升园区规划、产业层次、配套建设水平，力争6个省级开发区实现产值265亿元，完成固定资产投资460亿元，实现财政收入43亿元。支持市经济开发区、亳芜现代产业园区加快建设职教园、医疗器械产业园、药业机械产业园、云计算大数据产业园、高新技术产业园等“园中园”，谯城经济开发区争创国家级开发区，涡阳经济开发区加快建设光机电产业园，蒙城经济开发区加快建设汽车制造及零部件配套产业园，利辛经济开发区加快建设丝网服饰产业园。大力推进标准化厂房建设，6个省级开发区建设标准化厂房40万平方米以上。
　　加快振兴县域经济。强化产业支撑，大力发展特色经济、民营经济。在产业政策支持、政府投资引导基金投入、招商引资项目布局、重大项目建设等方面，加大对县域产业发展的倾斜力度，力争县域经济总量突破1000亿元、财政收入达100亿元。支持涡阳县重点发展装备制造、食品饮料加工等产业，蒙城县重点发展汽车制造及零部件生产、农机装备制造、食品加工等产业，利辛县重点发展食品及农产品加工、能源等产业，谯城区重点发展现代中药、食品饮料加工等产业，促进县域经济协调发展。各县区分别储备谋划项目200个以上、总投资规模不低于600亿元。
　　（五）突出城市能级提升，加快建设省际交汇区域中心城市
　　优化城市布局。编制市域空间综合规划，科学确定城市发展边界，认真做好城市规划与土地利用总体规划及各专项规划的衔接。加快教育、文化、体育、卫生以及其他公共服务设施规划编制，促进公共资源均等化配置。注重整体有序、联动开发，同步抓好老城区改造提升，加快高铁站片区和谯城区北部新城建设，推动市经济开发区和亳芜现代产业园区产城融合发展。
　　完善城市功能。着力提升中心城区集聚力和辐射力，市本级年内投资110亿元、实施城建项目303个，配套建设科教文卫体等公共服务项目83个、基础设施提升和园林绿化项目200个。加快建设日供水能力20万吨的城南地表水厂、市体育馆、市中心城区停车场等项目。实施引江济淮亳州段城区供水配套工程，启动建设输水管道和城南调蓄水库。加快推进海绵城市试点工程和综合管廊建设，改造老城区主次干道雨污分流管网20公里。完成11条路段河段夜景亮化工程。加大老城区拆迁改造力度，加快华佗广场二期、筛子市、柴家沟、灵津渡社区及周边地块等7个项目棚户区改造，推进陵西湖、涡河景观带建设，提升汤陵公园、曹操公园等建管水平，建成市科普公园和市防灾减灾科普馆，完成道东片区2号和3号地块2.3万亩土地、140万平方米房屋的征迁工作。加强三县县城建设，年度投资34.7亿元、实施37个重大基础设施项目，完善路网、水网、电网等基础设施，建成数字化城市管理平台，提高县城整体承载力和吸引力。
　　构建综合交通体系。启动交通建设大会战，建设贯通南北、连接东西的综合交通体系，年内投入37亿元。加快建设商合杭高铁亳州段，继续实施涡河高等级航道整治工程，积极推进亳州机场、亳蚌城际铁路、淮阜城际铁路、亳蒙高速、中心城区快速道路系统前期工作。完善城区交通网络，加快建设中心城区绕城快速通道、亳州北公交客运枢纽站、8个公交首末站及一批公交站台，开工建设高铁南站客运枢纽站和蒙城客运枢纽站，推进涡阳火车站升级改造。提升市域通畅能力，改建国省干线公路260公里以上，建成农村道路畅通工程2000公里以上。
　　（六）突出绿色生态发展，加快改善城乡生活环境
　　继续推进“地净、路畅、水活、天蓝、林拥城”工程。提高道路清扫保洁质量和市容市貌管理水平，全面实施“五车”信息化监管，实现市区数字城管全覆盖。加快实施中心城区快速路网工程、年内建成12公里，打通木兰路、柴胡路等4条断头路，加快建安路涡河隧道、汤王大道涡河隧道建设。实施凤尾沟三期、团结沟、龙凤新河等城区水系贯通治理工程。严格落实环境空气质量和大气污染防治工作“双考核”制度，启动安徽瑞福祥食品有限公司搬迁工作，加快市静脉产业园规划建设，认真做好秸秆禁烧工作，PM10和PM2.5年均浓度、主要污染物减排、空气优良天数完成省下达任务。建成“林拥城”环城林带，新建城市绿道50公里，打造“城在林中、林在城中”的城市生态景观。
　　建设美丽乡村。认真落实美丽乡村、美丽集镇建设规划和中心村布点规划，推进2017年度43个乡镇政府驻地建成区整治工作，加强基础设施建设和公共服务配套，同步开展80个中心村建设和全市所有自然村环境整治工作。支持利辛县做好美丽乡村整县推进建设工作。立足中医药、白酒、生态农业等优势产业，规划建设5个产业特色鲜明、人文气息浓厚、生态环境优美的特色小镇，年内至少启动2个特色小镇建设。大力推进农村土地综合整治和采煤塌陷区综合整治工作，完成城乡建设用地增减挂钩项目1.5万亩，复垦利用工矿废弃地5000亩，补充耕地2万亩。
　　严守生态红线。完成人工造林7万亩，建设森林生态廊道480公里、生态农田林网92万亩，争创省级生态乡镇和省级生态村各3个。全面推行“河长制”，加强53条河流72个断面监测，西淝河断面水质改善至III类。强力推进水污染防治，实施东城海子等35个黑臭水体治理项目。强化畜禽养殖污染和农业面源污染治理，完成185家禁养区内养殖场搬迁关闭和60家以上规模化畜禽养殖场污染综合治理任务。加强饮用水源地保护，做好土壤污染防治工作。加快建设涡阳道源湿地公园、蒙城北淝河湿地公园、利辛西淝河湿地公园等湿地保护与恢复工程。加快国家新能源示范城市建设，推进秸秆综合利用产业化，农作物秸秆综合利用率87%。全面开展城乡环境综合治理，统筹推进农村清洁工程、“三线三边”环境治理工作，加快生活垃圾终端处理设施建设，推行城乡环卫一体化试点工作。
　　（七）突出内生动力激发，加快推进改革开放
　　深化供给侧结构性改革。坚定不移去产能，综合运用市场化法治化手段，处置“僵尸企业”，清理“僵尸合同”，严防已化解产能死灰复燃。加大房地产市场调控力度，适度增加住宅用地供应，着力提高货币化安置比例，促进房地产市场平稳健康发展。加强政府债务管控，积极争取地方政府债券转贷资金。鼓励利辛县抓住贫困县IPO绿色通道机遇，引进拟上市企业。继续落实好全面营改增和各项减税政策，降低制度性交易成本和要素成本。创新政府投融资机制，推广运用政府与社会资本合作PPP模式，鼓励社会资本参与重点项目建设。
　　推动重点领域和关键环节改革。大力推进“放管服”，动态调整权力、责任、行政收费、公共服务、中介服务“五个清单”，全面推开“双随机、一公开”模式，优化提升“互联网+政务服务”，最大程度方便群众和企业。深化商事制度改革，全面推进“五证合一、一照一码”和企业简易注销登记，加快构建事中事后监管体系。深化财税金融体制改革，加强财政预决算管理，盘活财政存量资金，搭建国地税一站式联合征收平台，推动政府融资平台市场化转型，规范民间投融资行为。深化国企国资改革，组建市本级国资运营管理公司，支持古井集团、建安集团做大做强，实施国有企业和事业单位公务用车制度改革。完善公共资源交易平台运行机制，推动“六统一联”落到实处。推进户籍制度改革，全面实施居住证制度，有序推进农业转移人口市民化。深化供销合作社综合改革。
　　提升对外开放水平。持续优化外贸发展环境，加大政策扶持力度，提升贸易便利化水平。推动外贸转型升级，加强外贸主体培育，强化外贸进出口骨干企业调度和服务，全年新增进出口实绩企业20家。着力优化进出口结构，支持中药提取物、食品饮料、健康养生等优势产品出口，鼓励企业引进国外先进技术、设备和原材料，支持有条件的企业“走出去”。大力引进外资企业，力争外商直接投资增长8%左右。主动参与淮河生态经济带建设分工与合作。深化与芜湖市的结对合作。积极参与“牵手世界的安徽”等重大展会，办好2017年国际（亳州）中医药博览会暨第33届全国（亳州）中药材交易会。
　　发展民营经济。按照“非禁即准”原则，全面放开投资领域，推进各项政策落地见效，大力扶持民营企业做大做强，实现民营经济增加值650亿元、增长10%，民营经济增加值占比提高1个百分点。加大对“双创”的财税支持和金融服务力度，重点扶持筑梦社区、青年创客空间发展，建设各类创业孵化基地和众创空间，鼓励老乡返乡创业、青年学子创业，新增私营企业6000家、个体工商户2万户以上。新创建省级中小企业公共服务示范平台2家、省级小微企业创业基地2个。用好“政企直通车”微信平台，穷尽办法、倾尽所能、依法依规帮助企业解决实际问题，增强民营企业家发展信心，让企业家专注创业、安心创业，构建“亲”“清”新型政商关系。
　　（八）突出“六个精准”，坚决打赢脱贫攻坚战
　　层层压实脱贫责任。认真落实“六看六确保”“四个全覆盖”工作要求，坚持满足扶贫工作一切需要，火力全开，适当宽裕，分级负责，各方协同，工作到户，责任到人，确保全面实现人脱贫、村出列、县区摘帽的目标。扎实推进精准扶贫精准脱贫，动态完善“四个清单”和贫困村贫困户帮扶措施大全，建立返贫预警机制，完善巩固脱贫后续计划和保障措施。按照“源头严把、过程严控、验收严格、纪律严厉”要求，建立核查、评估、退出和问责机制，压实县区、乡镇、村各级责任，确保脱贫结果真实可信、脱贫成效经得起检验。
　　深入实施脱贫攻坚八大工程。大力发展特色种养业扶贫，完成光伏扶贫项目建设。加快易地扶贫搬迁安置点建设，完成3745人搬迁任务。对未考上高中或大学的贫困家庭子女实现免费中等职业教育和“雨露计划”全覆盖，从根本上阻断贫困代际传递。落实健康脱贫基本医疗保障优惠政策，提升贫困群众医疗保障水平，破解因病致贫、因病返贫难题。加强基础设施建设，完成贫困村道路畅通工程、电网改造、高标准基本农田建设任务，改造贫困户危房15393户。
　　千方百计加大扶贫投入。市本级安排财政预算扶贫资金5.7亿元，其中新增4.7亿元，将当年财政收入增量的20%以上、涉农资金统筹整合出的40%以上、清理收回财政存量资金可统筹使用部分的50%以上，以及压缩出的行政办公经费全部用于扶贫工作。将建档立卡贫困户的扶贫标准提高到4310元。推进县区统筹整合使用财政涉农资金，发挥财政扶贫资金杠杆作用，多方撬动社会资本投入扶贫工作。
　　（九）突出民生保障，全力提升群众幸福指数
　　着力保障改善民生。全面完成省下达的民生工程任务。坚持就业优先，重点抓好返乡农民工进城就业，新增城镇就业3万人、转移农村富余劳动力7.2万人、进城就业农民工2.1万人。健全社会保障体系，实行全民参保登记，稳妥推进机关事业单位养老保险制度改革。城市低保标准与城镇居民人均消费性支出同比例提高，实现农村五保分散供养标准、低保标准、扶贫标准“三线合一”。加大农村留守儿童关爱保护力度，认真解决低收入人群的法律援助问题。建成并投入使用市精神病人福利中心、养老服务中心。继续实施农村饮水安全巩固提升工程，实现农村安全饮水村村通。
　　统筹发展各项事业。优先发展教育事业，提升学前教育发展水平，深化教师无校籍管理改革，实施乡村教师支持计划，推进义务教育均衡发展；年内在中心城区新建改扩建8所中小学、3所公办幼儿园，完成180个“村小提升工程”项目；启动亳州职业技术学院二期工程建设，建成亳州特教学校新校区，完成亳州中药科技学校、亳州幼儿师范学校二期工程建设。继续支持亳州学院建设有特色的地方高水平应用型本科高校，支持亳州职业技术学院创建地方技能型高水平大学。深化医药卫生体制综合改革，扎实推进“三医联动”，实现城乡居民基本医疗保险“六统一”；加快华佗中医院新院建设，确保年内投入使用；启动建设市第二人民医院、市妇幼保健计划生育服务中心；积极发展社会办医，年内建设二级及以上医院5家；开展第三届全市名中医评选活动；人口自然增长率控制在9.5‰以内。持续提升公共文化体育服务能力，加快建设公共文化服务设施，建成并投入使用市“五位一体”公共活动中心，开工建设市广电中心；繁荣地方文艺创作，传承发展非物质文化遗产，广泛开展送戏下乡、送戏到敬老院和城市文化广场活动。建设城区15分钟健身圈，办好第三届中国（亳州）国际健身气功博览会，在全国推广校园五禽戏，筹办国际半程马拉松比赛等各种赛事活动。
　　积极创新社会治理。推进“七五”普法工作，加强公共法律服务体系建设。落实“三位一体”信访工作责任制和领导干部开门接访、带案下访、重点约访工作制度，努力打造“阳光信访、责任信访、法治信访”。强化精神文明创建，建成安徽好人馆亳州馆，抓好中国好人、道德模范评选宣传活动，全面提升公民道德水平。健全食品药品安全监管网络，提高监管保障能力，扎实推进食品药品安全城市创建工作。扎实开展“安全生产隐患排查治理年”活动，深入推进重点行业和领域专项整治，提升消防火灾防控水平，清理危化品运输车辆，取缔非法的混凝土沥青搅拌站、码头港口、小型加气站，认真排查农村枯井、河塘、自建房等各类隐患，确保安全隐患整改率达98%以上。加快视频数据监控系统建设，推进市县乡村四级综治维稳信访工作中心（站）规范化、实战化。健全完善社会治理体系，加强矛盾纠纷排查化解，完善社会稳定风险评估制度，最大限度预防和减少社会矛盾。加强互联网安全管理，加大治安突出问题专项整治，严密防范和依法打击各类违法犯罪行为，全力建设更加稳定、和谐、有序的“平安亳州”。加强国防动员、国防教育和民兵预备役建设，推进军民融合深度发展，争创新一轮省级双拥模范城（县）。强化统计管理，完成第三次农业普查。开展第十届村委会换届选举。统筹抓好外事、侨务、对台、民族宗教、防震减灾、应急管理、公积金管理、无线电管理和机关事务管理工作，推动档案、地方志、红十字、残疾人、妇女儿童、新闻出版、文物保护和关心下一代等事业健康发展。
　　（十）突出自身建设，着力提升政府工作水平
　　站在新起点，面对新期待，我们一定牢记宗旨、不辱使命，夙夜在公、勤勉工作，不忘初心、继续前进。坚持讲看齐、见行动。牢固树立“四个意识”特别是核心意识、看齐意识，始终在思想上政治上行动上与以习近平同志为核心的党中央保持高度一致，确保党中央、国务院、省委、省政府和市委的各项决策部署落地生根。坚持依法行政。自觉接受市人大及其常委会的法律监督和工作监督，自觉接受市政协的民主监督，自觉接受社会和舆论监督，主动倾听各民主党派、工商联、无党派人士意见建议，充分发挥工会、共青团、妇联等人民团体重要作用，切实做好政府立法和政务公开工作，认真办理人大议案建议和政协委员提案，确保政府工作始终在法治轨道上运行。坚持为民执政。坚持以人民为中心的发展思想，把改善民生福祉作为政府工作的出发点和落脚点，集中力量做好普惠性、基础性、兜底性民生建设，切实解决好人民群众关心关注的脱贫攻坚、就业创业、子女教育、看病就医、健康养老等现实问题，竭尽全力让亳州百姓过上更加美好幸福的生活。坚持务实勤政。强化责任担当，弘扬实干作风，铆足干劲、增强闯劲、保持韧劲，把所有心思用在谋发展上，把全部精力用在干事业上，立下军令状，明确作战图，咬紧牙关、攻坚克难，确保干一件、成一件、积小胜为大胜。坚持廉洁从政。严格执行党内政治生活的若干准则、党内监督条例、廉洁自律准则、纪律处分条例，认真落实中央八项规定精神和省市“三十条”规定，全面加强政府系统党风廉政建设，认真落实主体责任，切实履行“一岗双责”，持续强化审计监督，始终保持惩治腐败高压态势，严肃查处侵害群众利益的不正之风和腐败问题，着力打造忠诚干净担当的干部队伍。
　　各位代表，事业任重道远，责任重于泰山。让我们在省委、省政府和市委的坚强领导下，团结带领全市人民，凝心聚力，奋发进取，加快跨越赶超，决胜全面小康，为“深入践行五大发展理念，奋力走在皖北振兴前列”而努力奋斗，以优异成绩迎接党的十九大胜利召开！
